# Blood vessels and cancer much more than just angiogenesis

**DOI:** 10.1038/cddiscovery.2015.64

**Published:** 2015-12-21

**Authors:** F Pezzella, A L Harris, M Tavassoli, K C Gatter

**Affiliations:** 1 Radcliffe Department of Medicine, Nuffield Division of Clinical Laboratory Science, University of Oxford, John Radcliffe Hospital, Oxford, UK; 2 Molecular Oncology Laboratories, Department of Medical Oncology, Wheatherall Institute of Molecular Medicine, University of Oxford, Oxford, UK; 3 Department of Molecular Oncology, King’s College London, London, UK

The availability of oxygen and nutrients supplied by the vasculature is crucial for both normal and neoplastic cells, however, the nature of the intratumour vessels and their relationship to the neoplastic cells has been debated for centuries.

The introduction of the concept of angiogenesis in tumours, i.e. formation of new vessels, goes back as far as 1787^1^ and in 1939 Ide *et al.*^2^ described that tumour implants in the ears of rabbits were accompanied by formation of new capillaries leading to the idea that angiogenesis is necessary to support tumours. Yet, at the same time several histopathologists maintained the point that both pre-existing and newly formed vessels were co-existing in tumours and, sometime, only pre-existing vessels could be observed.^3^ However, it was not until 30 years later that the subject started to be systematically studied by Judah Folkman^4^ following the path indicated by Ide.

Folkman’s pioneering work on cancer and its relationship to vessels lead him and many others to describe the response of tumours to hypoxia as leading to angiogenesis, and therefore allowing cancer growth.^5^ The biological pathways underlying these processes were assumed to be pretty much common to all the tumours leading to the concept of ‘Inducing angiogenesis’ as an Hallmark of Cancer.^6^ On this assumption it was also deduced that by targeting the tumour-associated newly formed vessels, the cancer cells deprived of their newly formed vascular bed would die or, at least, would stop growing and would become dormant.^7^

However, an increasing body of evidence, initially from histopathological studies and subsequently from animal models and clinical trials, has uncovered an added layer of complexity: the possibility that some primary and metastatic tumours can develop and progress in the absence of angiogenesis, by exploiting the pre-existing vasculature.^8^ Surprisingly, tumours growing in this way have been described in the past (Figure 1), but then forgotten.

The discovery that, alongside purely nonangiogenic tumors, there is a large number of cases containing both pre-existing vessels and newly formed ones,^9^ shows that ‘inducing angiogenesis’ is not an essential Hallmark of Cancer and lack of angiogenesis is far from being an exception.

This observation has also raised the question of the relative importance of angiogenesis versus pre-existing vessels in the high proportion of cancers containing both: therefore, in a large number of tumours, we can not be sure of how much angiogenesis and how much pre-existing vessels are contributing to tumour growth. These findings are therefore seriously questioning the idea that Folkman’s postulates are still largely valid.^10^

The best characterization so far of this type of growth is in malignant lesions of lung, liver and brain cancer. The non-angiogenic growth implies that as these tumours grow without the pathways of classical angiogenesis, they must have a different biological setting, so a new chapter in cancer biology is now in front of us. Among the new questions now raised, there are four main ones.

First, as these tumours, either primary or metastatic, grow and experience hypoxia why is angiogenesis not triggered?^11,12^ It is likely that this is specific to their biology than failure to respond to hypoxia. Attempts to map the pathways underlying the biological behaviour of such tumours suggest that metabolic reprogramming with a switch towards oxidative phosphorylation, under the control of selected heat-shock proteins, oncogenes and tumour-suppressor genes, could be one of the key issues.^11,13^

The second key question is how the cancer cells interact with and co-opt the vessels and how this process varies in different organs. Only a few studies, most focused on the brain, have so far addressed this issue. In primary brain carcinoma, cells are directed towards the vessels by the bradykinin signalling pathways,^14^ whereas metastatic cells instead accumulate around the vessels after extravasation and they are protected from apoptosis by neuroserpin.^15^ Adhesion of the malignant cells to the vessels can be mediated by L1CAM,^15^ beta1 integrin^16^ and/or connexins establishing gap junctions.^17^ In some of these models, the co-opted vessels eventually regress, following overexpression of angiopoietin 2 and ultimately angiogenesis is triggered.^18,19^

Some early data are also emerging to support co-option in the lung. In a recent study,^20^ Szabo *et al.* for the first time investigated in mouse models the anatomical interaction between cancer cells and the lung vessels during co-option. By employing confocal and electron microscopy the authors show that the metastatic cells first accumulate, after extravasation, within the alveolar wall to subsequently enter the airspace. Once there the metastases expand, as the malignant cells spread from one alveolar space to another as previously described.^3^ When the metastasis expands, some carcinoma cells appear to re-enter the alveolar wall positioning themselves between the endothelium and the epithelium. In this location, the metastatic cells split the basal membrane and anchor themselves to both the endothelial and the epithelial side. This is in broad agreement with the observed capacity that malignant cells have in the brain to anchor themselves to the basal membrane by L1CAM^15^ or beta1 integrin.^16^ The epithelial cells eventually detach from their basal membrane and undergo fragmentation. There is a different picture in the liver where the cancer cells exploit the liver blood flow by replacing the hepatocytes,^21^ but how this happens remains so far unclear.

Third, the canonical angiogenesis pathway hypothesized that neoplastic cells switch early on to angiogenic status and than cancer progression follows.^7^ Nevertheless, in contrast to this theory, non angiogenic metastases can occur in patients with angiogenic primary tumours.^22^ Whether this is owing to selection of nonangiogenic clones or possibility for a cell to switch back to a non-angiogenic setting, remains to be established.

Fourth, as some first studies on metastases show that co-option can be associated with inhibition of apoptosis,^15^ the question arises on how apoptosis and vascular co-option are related. Is this critical to the establishment of metastases, even independently of the subsequent vascular arrangements?

Clinically, the ability to identify vascular co-option in cancer is relevant to treatment for two main reasons: first is that it may be one of the causes of resistance to antiangiogenic drugs, and the identification of vascular co-option will help to distinguish which tumours are more likely to benefit and improve trial design. The second reason is that by unveiling the pathways dictating the nonangiogenic growth of cancer cells new targets for treatment could be discovered.

We are therefore once again spectators of what Thomas Huxley called ‘The great tragedy of science—the slaying of a beautiful hypothesis by an ugly fact.’

## Figures and Tables

**Figure 1:**
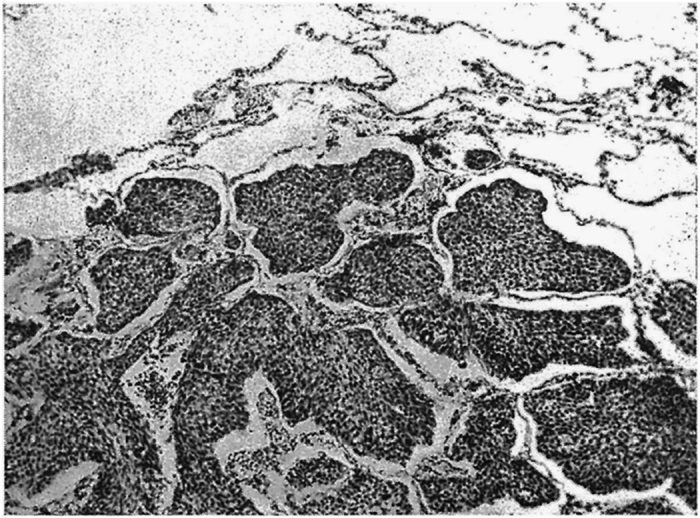
Nonangiogenic lung metastases described as ‘Intra-alveolar spread of a metastatic carcinoma of bladder in lung’ in 1948 ‘Pathology of Tumours’ by Rupert Willis.^23^
